# Atractylodin Produces Antinociceptive Effect through a Long-Lasting TRPA1 Channel Activation

**DOI:** 10.3390/ijms22073614

**Published:** 2021-03-31

**Authors:** Hirosato Kanda, Yanjing Yang, Shaoqi Duan, Yoko Kogure, Shenglan Wang, Emiko Iwaoka, Miku Ishikawa, Saki Takeda, Hidemi Sonoda, Kyoka Mizuta, Shunji Aoki, Satoshi Yamamoto, Koichi Noguchi, Yi Dai

**Affiliations:** 1 Department of Pharmacy, School of Pharmacy, Hyogo University of Health Sciences, Kobe 650-8530, Japan; kanda@huhs.ac.jp (H.K.); yj_yang2020@163.com (Y.Y.); r170037d@huhs.ac.jp (S.D.); y-kogure@huhs.ac.jp (Y.K.); wangshl@bucm.edu.cn (S.W.); miiwaoka@huhs.ac.jp (E.I.); ph15010@std.huhs.ac.jp (M.I.); ph15082@std.huhs.ac.jp (S.T.); ph15076@std.huhs.ac.jp (H.S.); ph16133@std.huhs.ac.jp (K.M.); aoki@huhs.ac.jp (S.A.); syamamot@huhs.ac.jp (S.Y.); 2 Department of Anatomy and Neuroscience, Hyogo College of Medicine, Nishinomiya 663-8501, Japan; noguchi@hyo-med.ac.jp; 3 Traditional Medicine Research Center, Chinese Medicine Confucius Institute at Hyogo College of Medicine, Nishinomiya 663-8501, Japan; 4 Department of Pathophysiology, Shenyang Medical College, Shenyang 110034, China; 5 School of Acupuncture-Moxibustion and Tuina, Beijing University of Chinese Medicine, Beijing 100029, China

**Keywords:** atractylodin, transient receptor potential ankyrin-1 (TRPA1), pain, dorsal root ganglion, QX-314

## Abstract

Atractylodin (ATR) is a bioactive component found in dried rhizomes of *Atractylodes lancea* (AL) De Candolle. Although AL has accumulated empirical evidence for the treatment of pain, the molecular mechanism underlying the anti-pain effect of ATR remains unclear. In this study, we found that ATR increases transient receptor potential ankyrin-1 (TRPA1) single-channel activity in hTRPA1 expressing HEK293 cells. A bath application of ATR produced a long-lasting calcium response, and the response was completely diminished in the dorsal root ganglion neurons of TRPA1 knockout mice. Intraplantar injection of ATR evoked moderate and prolonged nociceptive behavior compared to the injection of allyl isothiocyanate (AITC). Systemic application of ATR inhibited AITC-induced nociceptive responses in a dose-dependent manner. Co-application of ATR and QX-314 increased the noxious heat threshold compared with AITC in vivo. Collectively, we concluded that ATR is a unique agonist of TRPA1 channels, which produces long-lasting channel activation. Our results indicated ATR-mediated anti-nociceptive effect through the desensitization of TRPA1-expressing nociceptors.

## 1. Introduction

**Figure 1:**
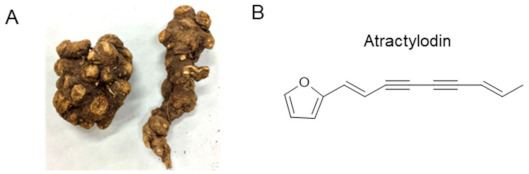
Source and structure of atractylodin (ATR). (**A**) Rhizomes of *Atractylodes lancea* De Candolle. (**B**) Chemical structure of ATR.

Atractylodin (ATR) is a polyethylene alkyne, a bioactive component of the dried rhizomes of *Atractylodes lancea* De Candolle (AL) (Figure 1A,B). AL has been used in traditional Chinese and Japanese medicine for the treatment of digestive diseases [1]. AL and ATR have been reported to improve intestinal inflammation and delayed intestinal motility in animal models of digestive disorders [2,3]. In addition to digestive diseases, herbal prescriptions containing AL, such as Ninjututo, Keishikajutsubuto, and Daibofuto, have often been used for pain management [4,5,6,7]. Although AL has accumulated empirical evidence for the treatment of pain in rheumatoid arthritis [7,8,9], its molecular mechanism remains unclear. 

Recent pharmacological studies on ATR have revealed that ATR activates the growth hormone secretagogue receptor, the ghrelin receptor, and promotes gastric emptying as well as stimulates food uptake [10]. In addition to its effect on the gastrointestinal system, ATR has been reported to possess various pharmacological activities, including anti-inflammatory, anti-cancer, anti-rheumatoid arthritis, and hepatoprotective effects [2,11,12,13]. To the best of our knowledge, the pharmacological effects of ATR on the sensory nervous system have not yet been investigated.

The transient receptor potential ankyrin-1 (TRPA1) is a nonselective cation channel that is predominantly expressed in a subset of nociceptive sensory neurons [14]. The main characteristic of the TRPA1 channel is that it acts as a sensor for a wide variety of chemical compounds, including environmental toxins, natural products, irritants, and endogenous reactive mediators [15]. These chemicals activate the channel in two different ways: covalent or non-covalent activation. Allyl isothiocyanate (AITC), acrolein, cinnamaldehyde, and formaldehyde are covalent agonists that act on the intracellular N-terminal domain of the channel [16,17]. In contrast, acids, Ca^2+^, carvacrol, clorimazole, propofol, and GNE551 act as non-covalent agonists [18,19,20]. A recent report suggested that based on covalent or non-covalent agonists, there were differences in activation of TRPA1 channels [20]. 

In this study, we examined the effects of ATR on the TRPA1 channel by comparing its activation properties with those of AITC. We further explored whether ATR has an antinociceptive effect. We observed that ATR acted as a TRPA1 agonist and produced long-lasting channel activation. Systemic application of ATR inhibited AITC-induced nociceptive responses in a dose-dependent manner. Co-administration of ATR and QX-314 increased the noxious heat threshold in vivo. Thus, we identified ATR as a unique agonist of TRPA1 channels, resulting in an antinociceptive effect. We further suggest that ATR may be useful in alleviating pain in clinical scenarios.

## 2. Results

### 2.1. ATR Induces a Long-Lasting TRPA1 Channel Activation

**Figure 2:**
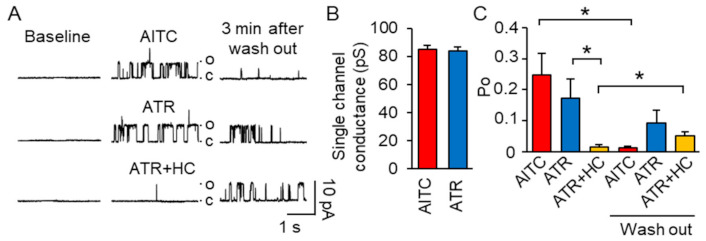
Bath application of atractylodin induced long-lasting TRPA1 channel activation in the hTRPA1 transfected HEK cells. (**A**) Sample traces illustrate TRPA1 single-channel currents induced by AITC (top, *n =* 4), ATR (middle, *n =* 6), and ATR + HC (bottom, *n =* 6) on the hTRPA1 expressing HEK293 cell recorded by cell-attached configuration. Holding potential was 60 mV. (**B**) Bar graph shows single-channel conductance. (**C**) Summary of the open probability of TRPA1 channel before and after washing out of AITC, ATR, or ATR+HC. Data represent mean ± standard error of mean, * *p* < 0.05, one-way analysis of variance with the Tukey post hoc test or Student’s *t*-test. AITC: allyl isothiocyanate; ATR: atractylodin; HC: HC-030031: Po: open probability.

To detect the effect of ATR on the TRPA1 channel, we performed a single-channel analysis on the cell membrane of hTRPA1-transfected HEK293 cells. In the cell-attached configuration, we applied AITC, a TRPA1 channel agonist, through bath solution. Bath application of 100 μM AITC evoked TRPA1 channel activation, which diminished 3 min after washout (Figure 2A). The single-channel conductance of the hTRPA1 channel was about 84.9 ± 2.8 pS (*n* = 4) at +60 mV (Figure 2B), consistent with a previous report [21]. Similarly, bath application of 10 μM ATR also evoked single-channel activities with a channel conductance of 84.0 ± 2.6 pS (*n* = 6) (Figure 2B). In addition, channel activity was completely blocked by HC-030031 (HC), a selective TRPA1 channel antagonist (Figure 2A). These data indicate that ATR passes through the cell membrane to activate the TRPA1 channel from the inside of the cell. Interestingly, unlike AITC, ATR induced a long-lasting channel activation, which continued beyond 3 min after washout (Figure 2A). To characterize channel activation, we analyzed the open probability of the TRPA1 channel before and after washout of AITC, ATR, or ATR+HC. AITC activated the TRPA1 channel with an open probability of 0.25 ± 0.08, which significantly decreased to 0.012 ± 0.01 (*p* < 0.05) 3 min after washout. ATR also activated the channel with an open probability of 0.17 ± 0.06; however, the value did not change significantly after washout (0.094 ± 0.04, *p* = 0.34) (Figure 2C). Interestingly, although HC significantly inhibited ATR-induced channel activity, the TRPA1 channel was activated after washing out HC without any additional ATR application. The open probability in the presence of HC was 0.015 ± 0.01, and it significantly increased to 0.05 ± 0.01 after washout (*p* < 0.05, Figure 2C).

**Figure 3:**
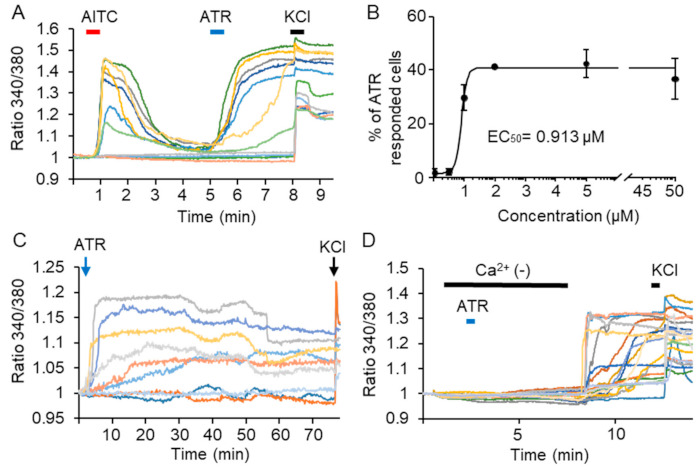
Bath application of atractylodin induced a long-lasting calcium response in the dorsal root ganglion neurons. (**A**–**D**) Calcium imaging analysis with rat primary cultured dorsal root ganglion neurons. Ratiometric measurement of Fura-2AM was performed to analyze cytosolic Ca^2+^ levels. Calcium response from different cells is shown in different colors. The calcium responses induced by bath application of 100 μM AITC followed by 5 μM ATR (**A**), dose–response curve of ATR (*n* = 3 in each group) (**B**), the long-lasting effect of ATR (**C**), and the effect of the calcium-free solution on ATR-induced calcium response (**D**). AITC: allyl isothiocyanate; ATR: atractylodin.

TRPA1 channels are expressed in sensory neurons and play an important role in the detection of pain. To confirm the effect of ATR on the channel in sensory neurons, we performed calcium imaging analysis on rat cultured-dorsal root ganglion (DRG) neurons. We first applied 100 μM AITC followed by 5 μM ATR to Fura-2AM loaded DRG neurons. Thirty-seven percent of the DRG neurons were activated by AITC (29/73 cells), and 96.6% of AITC-activated neurons showed a calcium response to 5 μM ATR (28/29 cells, *n* = 4, Figure 3A). We also generated a concentration response curve for ATR in the DRG neurons, and the EC_50_ value was estimated to be 0.913 μM (Figure 3B). Since single-channel analysis indicated that ATR had induced long-lasting TRPA1 channel activation, we tested whether ATR could induce a long-lasting calcium response in the DRG neurons. Interestingly, short-term ATR treatment for 30 s induced a long-lasting calcium response in the DRG neurons, which prolonged for more than 1 h (52 cells, *n* = 3) (Figure 3C). Although this calcium response was completely blocked by the calcium-free bath solution, a response was observed immediately after the infusion of a normal bath solution containing calcium ions (55 cells, *n* = 3) (Figure 3D). These results suggest that ATR induces long-lasting activation of the DRG neurons. 

**Figure 4:**
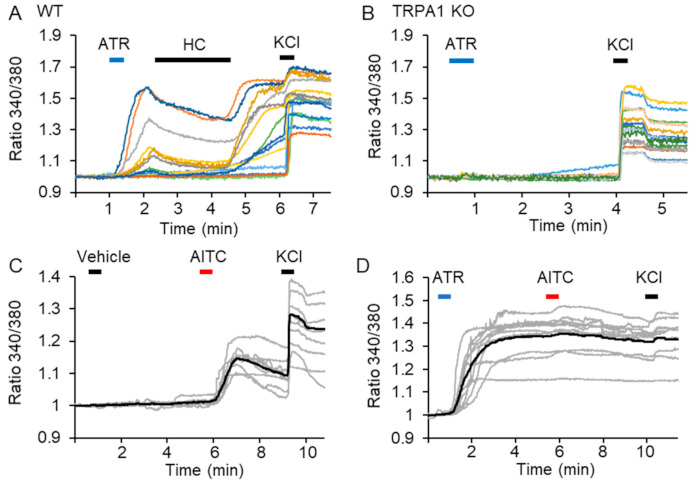
Atractylodin (5 μM) selectively activated transient receptor potential ankyrin-1 (TRPA1) channel in dorsal root ganglion (DRG) neurons. (**A**,**B**) Calcium imaging analysis with primary cultured DRG neurons from wild-type (**A**) and TRPA1 KO mice (**B**). Ratiometric measurement of Fura-2AM was performed to analyze cytosolic Ca^2+^ level. Calcium response from different cells is shown in various colors. The effect of HC-030031 on the ATR induced calcium response on cultured DRG neurons of WT mice (63 cells, *n* = 3) (**A**) and the effect of ATR on cultured DRG neurons from TRPA1 KO mice (95 cells, *n* = 3) (**B**). (**C**,**D**) Calcium imaging analysis with rat primary cultured DRG neurons. Calcium response from different cells is shown in gray lines, and the average response is shown in the black line (12 cells: C; 13 cells: D). ATR: atractylodin; HC: HC-030031; WT: wild-type; KO: knockout.

Since the DRG neurons express many ion channels, including transient receptor potential (TRP) channels, on their cell membrane, we confirmed whether ATR-induced long-lasting activation occurs only through TRPA1 channel activation. We found that ATR-induced long-lasting activation was attenuated by HC treatment. Consistent with single-channel analysis, these DRG neurons showed reactivation after washing out the HC (Figure 4A). Moreover, ATR (5 μM) did not induce any calcium response in the cultured DRG neurons of TRPA1 knockout (KO) mice (0%, 0/95 cells), (Figure 4B). These results indicate that 5 μM ATR selectively activates TRPA1 channels and causes a long-lasting activation in the DRG neurons.

We further examined whether the long-term activation of the TRPA1 channel could affect the sensitivity to subsequent AITC in the DRG neurons. Unlike the bath application of AITC-induced calcium response following vehicle treatment (Figure 4C), we did not observe any subsequent TRPA1 channel activation by AITC application during ATR-mediated long-lasting activation (Figure 4D). These results indicated that TRPA1 channel could get desensitized subsequent to induction by TRPA1 agonist during long-lasting channel activation.

### 2.2. Intraplantar Injection of ATR Induces Moderate But Prolonged Nociceptive Behaviors

**Figure 5:**
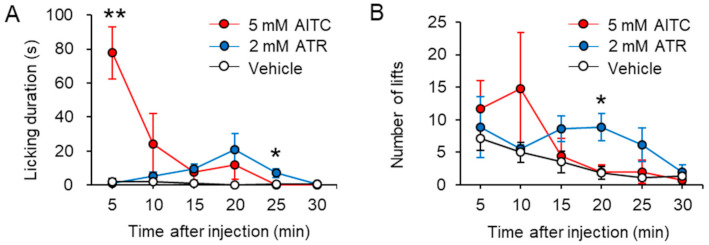
Intraplantar injection of atractylodin induced moderate and prolonged nociceptive behavior in rats. Licking duration (**A**) and number of lifts (**B**) induced by intraplantar injection of 5 mM AITC (*n* = 5) and 2 mM ATR (*n* = 6) over time (post injection). Data represent mean ± standard error of mean, * *p* < 0.05, ** *p* < 0.01 (AITC vs. ATR at the corresponding time point), one-way analysis of variance with the Tukey post hoc test. AITC: allyl isothiocyanate; ATR: atractylodin.

Based on our results of single-channel and calcium imaging analyses, we concluded that ATR caused long-lasting activation of TRPA1 in expressing cells. Since the TRPA1 channel is a well-known pain receptor, we injected ATR or AITC intraplantar and assessed the nocifensive behavior (licking and lifting) in rats. We observed that intraplantar injection of AITC induced intensive licking behavior, and the time of licking peaked at 5 min after the injection (Figure 5A). In contrast, ATR did not produce any licking behavior within 5 min. Instead, animals started showing small instances of licking behaviors 10 min after ATR injection, which was later significantly compared to those after AITC injection (Figure 5A). Consistent with the result of the licking behavior, we found that intraplantar injection of AITC induced lifting behavior immediately, whereas the same with ATR was observed much later (15 min after the injection) (Figure 5B). These results indicated that ATR did not provoke transient intensive, but moderate and prolonged nociceptive behaviors compared to AITC.

### 2.3. Systemic Application of ATR Attenuates AITC-Induced Nociceptive Behaviors Dose-Dependently

**Figure 6:**
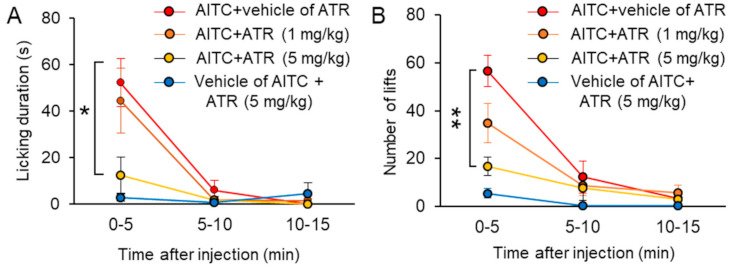
Systemic administration of atractylodin-attenuated AITC-induced nociceptive behavior in rats with dose-dependency. (**A**,**B**) Time course of nociceptive behavior induced by intraplantar injection of 5 mM AITC or vehicle of AITC following intraperitoneal injection of different doses of atractylodin (1 mg/kg, orange; 5 mg/kg) or vehicle of ATR (blue). The line graphs showing licking duration (**A**) and numbers of lifts (**B**) over time (post injection of AITC). Data represent mean ± standard error of mean, * *p* < 0.05, ** *p* < 0.01 (AITC + vehicle of ATR vs. AITC + ATR at 0–5 min, one-way analysis of variance with the Tukey post hoc test). AITC: allyl isothiocyanate; ATR: atractylodin.

Although ATR induced a long-lasting calcium response (>1 h), nociceptive behavior did not prolong in the in vivo experiments. It is known that capsazepine (CPZ), a TRP vanilloid-1 (TRPV1) channel antagonist, acts as an agonist of the TRPA1 channel, and systemic application of CPZ causes systemic TRPA1 inactivation [22]. In this set of experiments, we hypothesized that systemic ATR causes an analgesic effect through inactivation of the TRPA1 channel by long-lasting channel activation. To test our hypothesis, we pretreated rat with ATR intraperitoneally 20 min before the assessment of nociceptive behavior following intraplantar injection of AITC. AITC evoked intense licking behavior with licking duration of 52.2 ± 10.4 s, within 5 min after injection in the vehicle pre-injected rat (*n* = 9). Intraperitoneal injection of ATR (5 mg/kg) significantly inhibited AITC-induced licking duration to 12.3 ± 7.7 s, (*n* = 6, *p* < 0.05) within 5 min post AITC injection (Figure 6A), but this effect was not pronounced with administration of ATR (1 mg/kg). Further, pre-application of 5 mg/kg ATR significantly reduced the number of lifts from 56.7 ± 6.6 (*n* = 9) to 16.7 ± 4 (*n* = 6, *p* < 0.01) at 0.5 min after AITC injection (Figure 6B). The same value in the case of pre-application of 1 mg/kg ATR was 34.8 ± 8.2, (*n* = 6, *p* = 0.057 from vehicle control). Notably, 5 mg/kg ATR alone did not induce nociceptive behavior. 

### 2.4. Co-Application of ATR and QX-314 Increases Noxious Heat Threshold

**Figure 7:**
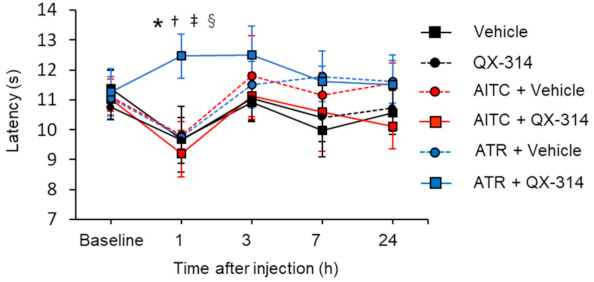
Time course of changing of noxious heat threshold by Hargreaves test after co-application of atractylodin and QX-314 in mice hind paw. Injections in the mice hind paw of different arms were as follows: 20 μl of vehicle (*n* = 6), 2% QX-314 (*n* = 6), 5 mM AITC + vehicle (*n* = 6), 5 mM AITC + 2% QX-314 (*n* = 6), 2 mM ATR + vehicle (*n* = 7), and 2 mM ATR + 2% QX-314 (*n* = 6). Data represent mean ± standard error of mean, * *p* < 0.05 (vehicle vs. ATR + QX-314), † *p* < 0.05 (AITC + QX-314 vs. ATR + QX-314), ‡ *p* < 0.05 (ATR + vehicle vs. ATR + QX-314), § *p* < 0.05 (QX-314 vs. ATR + QX-314), and two-way analysis of variance with the Fishers’s LSD post hoc test. AITC: allyl isothiocyanate; ATR: atractylodin.

QX-314 is a membrane-impermeable sodium channel blocker that has been shown to enter into the intracellular through the activating TRPV1 channels and by blocking the sodium channel intracellularly [23,24]. Based on the pore dilation of the TRPA1 channel, it has been suggested that the channel can also mediate the entry of QX-314 [25]. However, co-application of QX-314 and AITC failed to change heat or mechanical pain sensitivity [26]. It is known that TRPA1 is quickly inactivated following AITC application, which may prevent the entry of QX-314. Considering the long-lasting TRPA1 channel activation by ATR, we hypothesized that ATR may be more efficient in mediating the entry of QX-314. We employed the Hargreaves test to determine the noxious heat threshold of the hind paw of mice. We found that intraplantar injection of any of the following did not change the noxious heat threshold at 1 h after injection: vehicle, QX-314, AITC + vehicle, ATR + vehicle, or AITC + QX-314. The effect of AITC + QX-314 on the noxious heat threshold was consistent with that reported by a previous report [26]. In contrast, co-application of ATR and QX-314 significantly increased the threshold to 12.46 ± 0.73 s (*n* = 6) 1 h after intraplantar injection compared with ATR (9.77 ± 0.64 s, *n* = 7, *p* < 0.05), AITC (9.82 ± 0.46 s, *n* = 6, *p* < 0.05), and vehicle (9.68 ± 1.1 s, *n* = 6, *p* < 0.05), each being administered alone, Figure 7). 

## 3. Discussion

Plants contain various bioactive natural products, some of which, such as capsaicin, menthol, and cinnamaldehyde, have been accepted as selective agonists of TRP channels and activate the TRPV1, TRP melastatin 8, and TRPA1 channels, respectively. In this study, we first demonstrated that ATR, the main bioactive component of AL, produced long-lasting TRPA1 channel activation. Surprisingly, the ATR-mediated calcium response was significantly prolonged for more than an hour, following washout. The non-covalent agonists of TRPA1 channels, such as menthol and GNE551, are reversible and do not produce long-lasting channel activation [20]. In contrast, the reaction time of covalent agonists relies on their covalent binding affinity. AITC activates the TRPA1 channel through covalent modification of specific cysteine residues, which causes reversible activation [27]. Iodoacetamide and N-methyl maleimide are also covalent agonists of the TRPA1 channel but produce irreversible modifications of cysteine residues [28]. Our results showed that ATR induced prolonged TRPA1 channel activation compared to AITC. It is known that differences in chain size or ring of chemical structures influence the dissociation of the ligand from the TRPA1 channel [27]. ATR is an alkyne-polyacetylene containing a 2-nonyltetrahydrofuran skeleton, and we postulated that ATR might produce covalent activation of the TRPA1 channel, which could cause irreversible activation owing to its unique chemical structure. It would be interesting to examine whether ATR could directly modulate the cysteine residues of the TRPA1 channel and to study how ATR induces long-lasting TRPA1 channel activation.

The TRPA1 channel is activated by various chemical stimuli, and intraplantar injection of these chemical compounds produces nociceptive behavior in animals [29,30,31]. We found that intraplantar injection of ATR induced prolonged but moderate nociceptive behaviors compared with those of AITC. However, intense nociceptive responses were observed within 10 min of intraplantar injection of AITC. It has been reported that short-term application of AITC to TRPA1-expressing cells induces transient channel activation, and continuous application of AITC quickly desensitizes TRPA1 channels [32]. We believe that this transient nociceptive behavior induced by AITC may reflect the change in channel properties with time from the activation phase to the inactivation phase following AITC injection. The ATR-induced prolonged nociceptive behavior compared to AITC might be due to long-lasting TRPA1 channel activation.

Some herbal formulations containing AL are used for the treatment of pain. For instance, AL containing formulations Nijutsuto, Tokishakuyakusan, or Daibofuto have been prescribed as oral treatments for joint and shoulder pain [4,5,7]. In the present study, we found that ATR acted as a TRPA1 channel agonist, and intraplantar injection of ATR evoked moderate nociceptive behavior. An important finding was that although ATR produced long-lasting TRPA1 activation and continued for more than 1 h in vitro, moderate nociceptive behavior peaked at 20 min after injection and disappeared after 30 min in vivo (Figure 5). Accordingly, we postulated that long-lasting activation of the TRPA1 channel could cause desensitization of nociceptors. We also found that systemic application of ATR attenuated AITC-induced nociceptive behavior. It is known that CPZ acts as an agonist of TRPA1 channel, and systemic application of CPZ induces systemic TRPA1 inactivation. Interestingly, CPZ-treated animals have shown attenuated nociceptive behavior following AITC treatment [22]. In addition, it is known that compounds such as cinnamaldehyde, menthol, and camphor have bimodal effects on TRPA1 channels, and they serve either as activators or blockers depending on their concentration [33,34]. However, we did not observe a bimodal effect of ATR on TRPA1 channels at concentrations ranging between 50 nM and 50 µM. These results suggest that systemic ATR might interfere with TRPA1 channels following intraperitoneal injection, leading to an antinociceptive effect. It has never been clear whether oral administration of AL is for an anti-pain effect; however, it is assumed that ATR may play an important role by interrupting TRPA1 channels and subsequently desensitizing the TRPA1-expressing nociceptors. Further studies are needed to determine whether the anti-nociceptive effect of ATR relies on the TRPA1 channel in TRPA1 KO mice in vivo. 

Although the TRPA1 channel is activated by a variety of environmental irritants and is related to chemical-evoked pain, the contribution of the TRPA1 channel for detecting noxious mechanical and noxious cold pain is still controversial [35]. Previous studies have shown that QX-314 enters the cell through the TRP channels and increases mechanical, heat, and cold pain thresholds by blocking sodium channels in vivo [23,26,36]. Kobayashi et al. [35] reported that approximately 66.7% of TRPV1-expressing DRG neurons co-express TRPA1 channels, and 100% of TRPA1-expressing DRG neurons co-express TRPV1 channels. Therefore, blocking the TRPA1 expressing nerve terminal is assumed to increase the noxious heat threshold. In this study, we showed that although co-application of AITC and QX-314 failed to increase the thermal threshold (Figure 7), co-application of ATR and QX-314 significantly increased the threshold. These results might indicate that long-lasting TRPA1 channel activation could allow the entry of QX-314 into the intracellular region with higher efficiency than AITC. Notably, the amplitude of nociceptive sensation induced by ATR was not as strong as that induced by AITC, and ATR showed high efficiency in facilitating QX-314 entry into the cells. Since QX-314 requires a TRP channel agonist such as capsaicin to enter the intracellular region, agonist-evoked transient nociception immediately after the injection is crucial for clinical treatment. We suggest that ATR might be useful for obtaining enough QX-314 into the intracellular space without intense noxious sensation. It will be interesting to examine whether ATR has a higher efficiency for facilitating QX-314 entry in vitro.

In the present study, we have identified that ATR, the major bioactive component of AL, acts as a unique TRPA1 agonist and has bimodal effects on nociception. The molecular mechanism of antinociceptive effect of AL itself or herbal prescription containing AL has never been reported. Our findings may be important in understanding how AL relieves pain. In addition, we suggest that ATR/AL treatment may be helpful in clinical scenarios. 

## 4. Materials and Methods

### 4.1. Animals

Male Sprague–Dawley (SD) rats (6–8 weeks old) (Japan SLC Inc., Shizuoka, Japan) and C57BL/6 mice (7–12 weeks old) were used for experiments. TRPA1 deficient mice were provided by Makoto Tominaga (Okazaki Institute for Integrative Bioscience, NINS, Okazaki, Japan) and were originally from David Julius (UCSF, San Francisco, CA, USA) [37]. The animals were housed in a temperature-controlled room (24 °C) and maintained on a 12 h light/dark cycle. The experimental protocols were approved by the Hyogo University of Health Sciences Committee on Animal Research. All experimental procedures were performed in accordance with the NIH Guide for the Care and Use of Laboratory Animals.

### 4.2. Primary Culture of the DRG Neurons

The DRGs from SD rats were isolated and prepared as previously described [38]. Briefly, the DRGs were corrected and placed in ice-cold Earle’s balanced salt solution (EBSS). After removing the connective tissue surrounding the DRGs under the microscope, they were treated with 1.25 mg/mL collagenase in EBSS at 37 °C for 60 min. After dissociation of the DRG, the cell suspensions were plated onto poly D-lysine-coated coverslips. Cells were grown in EBSS with 10% fetal bovine serum (FBS), 2 mM glutamax (Thermo Fisher Scientific, Waltham, MA, USA), and 100 ng/mL of nerve growth factor in presence of penicillin and streptomycin. 

### 4.3. HEK293 Cell Culture and Transfection

HEK293 cells were maintained in Dulbecco’s modified Eagle’s medium (supplemented with 10% FBS, 2 mM glutamax, penicillin, and streptomycin) and transfected with 1 μg of human TRPA1 (hTRPA1) cDNA using lipofectamine 2000 (Thermo Fisher Scientific, Waltham, MA, USA). TRPA1 cDNA was a generous gift from Ardem Patapoutian (The Scripps Research Institute, La Jolla, CA, USA). To identify transfected cells, an enhanced green fluorescence protein reporter plasmid was also transfected at 0.1 μg.

### 4.4. Calcium Imaging Analysis

A total of 20 rats, three C57BL/6 mice, and three TRPA1 deficient mice were used for calcium imaging analysis. Twenty-four hours after dissection of the DRG neurons, they were loaded with 4 μM Fura-2AM (Nacalai Tesque, Kyoto, Japan) for 1 h at 37 °C. The coverslip was placed into the recording chamber, and a normal bath solution containing 140 mM NaCl, 5 mM KCl, 2 mM MgCl_2_, 2 mM CaCl_2_, 10 mM 4-(2-hydroxyethyl)-1-piperazineëthanesulfonic acid (HEPES), and 10 mM glucose at pH 7.4, adjusted with NaOH, was perfused. Depolarization of the DRG neurons was induced with 50 mM KCl in a normal bath solution. Ratiometric calcium imaging was performed using a fluorescence microscope (IX71, Olympus) equipped with a digital camera (C4742-80-12AG, Hamamatsu Photonics, Hamamatsu, Japan). Dual images (at 340 and 380 nm excitation) were collected every 1 s and analyzed using HCImage Acquisition software (Hamamatsu Photonics, Hamamatsu, Japan). A microscopic field containing 20–30 neurons was randomly selected under the 20X objective. 

### 4.5. Single-Channel Patch-Clamp Analysis

Cultured cells were perfused with a normal bath solution containing 145 mM NaCl, 5 mM KCl, 2 mM MgCl_2_, 2 mM CaCl_2_, 10 mM glucose, 10 mM HEPES, pH 7.3, and osmolarity 330 mOsm. For cell-attached recording, a standard bath solution was used as the recording electrode. A patch pipette of <5 MΩ was used for recording. Stated membrane potentials refer to the inner physiological side of the membrane. The experiment was performed with voltage-clamped holding at 60 mV. The single-cell currents were amplified using an Axopatch 200 B, filtered at 2 kHz, and sampled at 10 kHz using pClamp 10 (Molecular Devices, San Jose, CA, USA). 

Then, 100 μM AITC, 10 μM ATR, or 10 μM ATR + 10 μM HC-030031 were bath applied for 1 min after establishing cell-attached mode; thereafter, the chemical was washed off with normal bath solution. To confirm the channel activity of the TRPA1 channel, we tested 100 μM AITC at the end of the experiment and analyzed single-channel activities that responded to AITC. 

### 4.6. Assessment of Nociceptive Behaviors

Rats (weighing within 190–200 g) were acclimated to the experimental environment for three consecutive days before the behavior test. To evaluate nociceptive behaviors, the licking time and number of right hind paw lifting were measured every 5 min. Briefly, 30 μL of each of 5 mM AITC, 2 mM ATR, or vehicle (10% dimethyl sulfoxide (DMSO) and 5% Tween 20 in saline) were injected into the right hind paw with a 27-gauge needle to evoke nociceptive behaviors. To study the anti-nociceptive effect, ATR (1 or 5 mg/kg) or its vehicle (10% DMSO and 5% Tween 20 in saline) was intraperitoneally administered 20 min before the intraplantar injection of 5 mM AITC or its vehicle (10% DMSO and 5% Tween20 in saline).

To test the effect of QX-314, 20 µL of vehicle (10% DMSO and 5% Tween20 in saline), 5 mM AITC with/without 2% QX-314, or 2 mM ATR with or without 2% QX-314 was injected into the left hind paw of mice. The noxious heat threshold was assessed using the Hargreaves test (Ugo Basile, Varese, Italy). The voltage of the heat source was adjusted to yield a baseline latency ranging from 8 to 11 s, and the cut-off time was set to 20 s to avoid tissue damage. Paw withdrawal latency was calculated using the average of three consecutive measurements. Behavioral tests were performed under double-blind conditions.

### 4.7. Statistics

All statistical analyses were performed using GraphPad Prism software (version 7, LaJolla, CA, USA). Data are expressed as the mean ± SEM. Differences in values between each group were tested using one-way analysis of variance (ANOVA), followed by individual post hoc comparisons (Tukey’s post hoc test). Differences in values over time for each group were tested using a two-way ANOVA, followed by individual post hoc comparisons (Fisher’s PLSD). Pairwise comparisons (Student’s *t*-test) were used to assess differences in values between the two groups. Differences were considered statistically significant at *p* < 0.05.

## Data Availability

The data that support the findings of this study are available from the corresponding author upon reasonable request.

## Abbreviations

ATR	Atractylodin
AL	Atractylodes lancea De Candolle
AITC	Allyl isothiocyanate
ANOVA	Analysis of variance
CPZ	Capsazepine
DRG	Dorsal root ganglion
HC	HC-030031
TRPA1	Transient receptor potential ankyrin-1
TRPV1	Transient receptor potential vanilloid-1
